# Use of Yeast Mannoproteins by *Oenococcus oeni* during Malolactic Fermentation under Different Oenological Conditions

**DOI:** 10.3390/foods10071540

**Published:** 2021-07-04

**Authors:** Aitor Balmaseda, Laura Aniballi, Nicolas Rozès, Albert Bordons, Cristina Reguant

**Affiliations:** 1 Grup de Biotecnologia Enològica, Departament de Bioquímica i Biotecnologia, Facultat d’Enologia, Universitat Rovira i Virgili, c/Marcel·lí Domingo 1, 43007 Tarragona, Catalonia, Spain; aitor.balmaseda@urv.cat (A.B.); laura.aniballi@virgilio.it (L.A.); cristina.reguant@urv.cat (C.R.); 2 Grup de Biotecnologia Microbiana dels Aliments, Departament de Bioquímica i Biotecnologia, Facultat d’Enologia, Universitat Rovira i Virgili, c/Marcel·lí Domingo 1, 43007 Tarragona, Catalonia, Spain; nicolasrozes@urv.cat

**Keywords:** *Oenococcus oeni*, non-*Saccharomyces*, mannoproteins, malolactic fermentation, gene expression

## Abstract

*Oenococcus oeni* is the main agent of malolactic fermentation in wine. This fermentation takes place after alcoholic fermentation, in a low nutrient medium where ethanol and other inhibitor compounds are present. In addition, some yeast-derived compounds such as mannoproteins can be stimulatory for *O. oeni*. The mannoprotein concentration in wine depends on the fermenting yeasts, and non-*Saccharomyces* in particular can increase it. As a result of the hydrolytic activity of *O. oeni*, these macromolecules can be degraded, and the released mannose can be taken up and used as an energy source by the bacterium. Here we look at mannoprotein consumption and the expression of four *O. oeni* genes related to mannose uptake (*manA*, *manB*, *ptsI,* and *ptsH*) in a wine-like medium supplemented with mannoproteins and in natural wines fermented with different yeasts. We observe a general gene upregulation in response to wine-like conditions and different consumption patterns in the studied media. *O. oeni* was able to consume mannoproteins in all the wines. This consumption was notably higher in natural wines, especially in *T. delbrueckii* and *S. cerevisiae* 3D wines, which presented the highest mannoprotein levels. Regardless of the general upregulation, it seems that mannoprotein degradation is more closely related to the fermenting medium.

## 1. Introduction

Malolactic fermentation (MLF) is a biotransformation undergone in fermented beverages with low nutrient composition by different lactic acid bacteria (LAB) [1,2,3]. It is usually performed in wine and cider and results in an increase in pH values because of the decarboxylation of L-malic acid into L-lactic acid, an improvement of organoleptic wine characteristics and an increased microbiological stability [4,5,6].

As it occurs in fermented media, the fermenting microbiota—mainly yeasts that carry out alcoholic fermentation (AF)—have a significant impact on LAB and on the development of the MLF, usually carried out after AF [7,8,9,10].

As a result of the transformation of grape must into wine (or apple juice into cider), the media in which the LAB ferments will have low concentrations of nutrients, high acidity, and high concentrations of ethanol and sulphur dioxide. All these changes select the LAB most suited to this stressful environment, of which *Oenococcus oeni* is the best adapted species [1,11,12].

These two fermentative processes are usually inoculated with the species best adapted to each fermentation, *Saccharomyces cerevisiae* and *O. oeni* for AF and MLF, respectively [13]. Today there is increasing interest in the use of certain non-*Saccharomyces* yeasts—naturally occurring in the first AF stages—as culture starters [14]. Non-*Saccharomyces* are inoculated together with a selected *S. cerevisiae* strain to ensure completion of AF.

This vast group of yeasts known as non-*Saccharomyces* includes various species such as *Torulaspora delbrueckii*, *Metschnikowia pulcherrima*, *Starmerella bacillaris,* and others [15]. Taking those wines fermented with *S. cerevisiae* as a reference, these species are associated with different chemical modulations such as lowering ethanol concentration [16,17,18], reducing medium-chain fatty acids and sulphur dioxide concentration [18,19], and increasing wine volatile compounds and mannoprotein concentration [16,18,19,20,21], as well as other effects, such as modulating total and volatile acidity and glycerol concentration [14,22]. These modulations are usually related to better MLF performance, as there is a decrease in inhibitory compounds and an increase in stimulatory compounds for *O. oeni* [10,23].

Among other things, the increase in mannoprotein concentration, especially through the use of *T. delbrueckii*, is stimulatory for *O. oeni* in wine MLF [10,24,25]. Mannoproteins are found in yeast cell walls and are the main polysaccharide released during AF and aging over lees [26]. They are formed by up to 80%–90% of monosaccharides, mainly mannose and traces of glucose, and around 10%–20% of amino acid residues [26,27].

During the winemaking process they can be released from the yeast cell wall, especially during aging, as a result of the yeast autolytic process [20,27]. Mannoproteins seem to play an important role in MLF development by adsorbing the medium-chain fatty acids produced by the yeasts and phenolic compounds of grape must, and stimulating bacterial growth [25,28,29]. It has also been demonstrated that *O. oeni* has glycosidase and peptidase activities that enable the release of sugars and amino acids from mannoproteins and other macromolecules, thereby increasing the nutritional content and the survival of *O. oeni* in wine [29]. Mannose, which is released from yeast mannoproteins because of mannosidase activity by *O. oeni*, may be a phosphotransferase system (PTS) substrate, which may then be involved in the stimulation of LAB growth in the presence of yeast mannoproteins or yeast extracts [30].

The main function of PTS is to translocate sugars across a membrane with simultaneous phosphorylation but without involving the concentration gradient [30]. PTS consists of several components including the enzyme I (EI), the histidine phosphocarrier protein (HPr) used in the phosphorylation cascade, and the substrate-specific permeases (enzyme II). The *ptsI* and *ptsH* genes repectively encoding the general PTS proteins EI and HPr are highly conserved in the species. The enzyme II complex, which forms a mannose-specific permease, consists of two hydrophilic domains (domains A and B) and one or two hydrophobic integral membrane domains (domains C and D). The genes *manA* and *manB* are widely found in *O. oeni* strains, whereas *manC* is more variable. EI and HPr (*ptsI* and *ptsH*) work with all sugars for the development of the phosphorylation cascade. The enzyme II complex is more substrate specific but, apart from any preferred substrate such as mannose for *manA or manB*, it can also be active with other sugars such as glucose [31].

The possible use of the mannoproteins released from yeasts, including non-*Saccharomyces*, by *O. oeni* during MLF has not been thoroughly addressed. The aim of this paper is to evaluate the ability of *O. oeni* to utilize yeast mannoproteins in different fermentation media and to study the transcriptional response of mannose-related genes as possible indicators of mannose consumption.

## 2. Materials and Methods

### 2.1. Microorganisms

The yeast strains used were *T. delbrueckii* Viniferm NS-TD (Agrovin, Alcázar de San Juan, Spain) (TdViniferm), *T. delbrueckii* Zymaflore Alpha (Laffort, Bordeaux, France) (TdZymaflore), *M. pulcherrima* Flavia (Lallemand Inc., Montréal, QC, Canada) (MpFlavia), *S. cerevisiae* Viniferm-3D (Agrovin, Alcázar de San Juan, Spain) (Sc3D), and *S. cerevisiae* Lalvin-QA23 (Lallemand Inc.) (ScQA23). For MLF, strain *O. oeni* PSU-1 (ATCC BAA-331) was used. It is a reference strain with the genome fully annotated, and from whose sequence the primers used here in the transcriptional study were deduced. Yeasts were maintained on YPD plates (2% glucose, 2% bacto-peptone, 1% yeast extract, 2% agar, *w/v*, Panreac Química SLU, Castellar del Vallès, Spain) and the bacteria on MRSmf [32] plates, and all of them were stored at 4 °C.

### 2.2. Experimental Fermentations

*O. oeni* PSU-1 was inoculated in two media to carry out MLF in oenological conditions. First, *O. oeni* was directly inoculated in a wine-like medium (WLM). Second, AFs were performed with different yeast combinations in natural grape must, and then in the inoculated *O. oeni* in the obtained wines.

#### 2.2.1. Fermentations in Wine-Like Medium

They were performed in 50 mL tubes containing 50 mL of sterile WLM, which was prepared as in Bordas et al. [33] with half nitrogen composition (1.25 g/L of casamino acids and 1.25 g/L of peptone). This model wine had a concentration of around 110 mg N/L of yeast assimilable nitrogen, 2 g/L of L-malic acid, and a pH of 3.4. A stock solution (40 g/L) of the commercial mannoprotein extract Mannoplus (Agrovin, Spain) was prepared in WLM under sterile conditions and the appropriate volume was added to each wine to obtain final concentrations of 0, 100, 200, and 400 mg/L of mannoprotein extract. Each wine (50 mL) was then inoculated with *O. oeni* PSU-1 for a population of around 2 × 10^7^ cells/mL. These fermentations were carried out in triplicate and incubated statically at 20 °C. Samples were taken every 24 h to monitor the evolution of L-malic acid consumption and the bacterial population. This was calculated by plating samples on MRSmf and incubating at 27 °C in a 10% CO_2_ atmosphere for 7 days. MLF was considered finished when L-malic acid was below 0.1 g/L.

#### 2.2.2. Fermentations in Natural Grape Must

These were performed with natural concentrated Airén must (Mostos S.A., Tomelloso, Spain) diluted with sterile Milli-Q water to a density of 1080 ± 1 g/L [23]. The must was supplemented with 0.4 g/L of Nutrient Vit NatureTM (Lallemand Inc.) and the pH was adjusted to 3.6. The must was then sterilized using 0.1% (*v*/*v*) of dimethyl dicarbonate (Santa Cruz Biotechnology, Inc., Dallas, TX, USA) and stored overnight at 4 °C.

In order to undergo the fermentations, precultured yeasts in YPD liquid medium were inoculated for a population of 2 × 10^6^ cell/mL. In the case of non-*Saccharomyces*, after 48 h from the initial inoculation, ScQA23 was also inoculated for the same population. Fermentations were carried out statically in 500 mL of must, at 20 °C, and in triplicate. Every 48 h, density (Densito 30PX Portable Density Meter, Mettler Toledo, Spain) and yeast population were determined. YPD agar plates were used to calculate the total number of yeast cells present, and lysine agar medium (Oxoid LTD., Basingstoke, UK) was used to quantify the non-*Saccharomyces* yeasts, after incubation at 28 °C for 48 h. AF was considered finished when the sugar concentration was below 2 g/L. At this point the wines were centrifuged at 10,000× *g* for 5 min at 4 °C, filtered (0.22 µm Whatman, Thermo Fisher Scientific, Waltham MA, USA), and transferred to sterile 50 mL tubes. The wines were then inoculated with *O. oeni* PSU-1 for a population of 2 × 10^7^ CFU/mL and incubated in the same conditions as the AFs. These MLF were also carried out in triplicate. Samples were taken every 24 h to monitor the consumption of L-malic acid and the evolution of the bacterial population, as described above.

### 2.3. Wine Characterization

Wines were analyzed after AF and MLF. The pH was measured with a pH-meter MicropH 2002 (Crison-Hach Lange, L’Hospitalet, Spain) and various compounds (primary amino nitrogen (NOPA), NH_4_, acetic acid, citric acid, L-lactic acid, L-malic acid, D-lactic acid, and glucose + fructose) were analyzed with a multianalyzer Miura One (TDI SL, Gavà, Spain). The mannoprotein content of WLM before and after MLF was quantified using a D-mannose and D-glucose assay kit K-MANGL (Megazyme, Wicklow, Ireland) as described in Balmaseda et al. [34].

### 2.4. Sampling and RNA Extraction

Cell pellets of *O. oeni* were collected during the fermentations. In the case of WLM, samples were collected at 1, 5, and 8 days after inoculation, corresponding to 24 h, end of MLF, and three days after the end of MLF (post-MLF), respectively. Sampling of the natural grape fermentations was performed at the end of MLF. Cells of *O. oeni* grown in MRSmf for 2 days were also collected. In all cases, 50 mL of wine was centrifuged (4250× *g*, 15 min) at 4 °C. The supernatant was discarded, and the cell pellet was frozen with liquid nitrogen and stored at −80 °C until analysis. The cell pellet was then washed twice with 1 mL of sterile 10 mM Tris-HCl buffer at pH 8 by centrifugating at 2000× *g* for 5 min at room temperature. Afterwards, the cell pellet was resuspended in 200 μL of the same Tris-HCl buffer with 50 mg/mL of lysozyme (Roche Life Science, Mannheim, Germany) and incubated for 30 min at 37 °C. Finally, the RNA was purified using a High Pure RNA Isolation Kit Version 13 (Roche Life Science) following the manufacturer’s instructions. The RNA was stored at −80 °C until analysis.

### 2.5. RT-qPCR

The RNA samples were cleaned of contaminant DNA traces with a TURBO DNA-free Kit (Thermo Fisher Scientific) following the manufacturer’s instructions. Reverse transcription (RT) and real-time qPCR were performed following Olguín et al. [35] using QuantStudio 5 Real-Time PCR Systems (Thermo Fisher). Four genes (*manA*, *manB*, *ptsI,* and *ptsH*) related to mannose intake by *O. oeni* were evaluated [30]. In addition, another four genes (*dnaG*, *dpoIII*, *gyrA,* and *gyrB*) were evaluated as internal controls. Of these, *gyrA* and *gyrB* presented the least variation within conditions (data not shown) and were therefore used as reference genes in this experimentation. The primers used for all the genes studied can be found in Supplementary Table S1. The ∆∆Ct method [36] was used to calculate the relative expression of each gene. The expression of *O. oeni* after growing for 2 days in MRSmf was used as the reference condition in WLM. To study the differences in this metabolism through the use of non-*Saccharomyces* in natural wines, the expression of *O. oeni* in ScQA23 wine was used as a control condition.

### 2.6. Statistical Analysis

All the statistical analyses of the results were performed using the statistics software XLSTAT version 2020.2.3 (Addinsoft, Paris, France). The analysis of variance was carried out by ANOVA with a subsequent Tukey HSD test to determine significant differences between the samples. The confidence interval used was 95% and the statistical level of significance was set at *p* ≤ 0.05.

## 3. Results and Discussion

### 3.1. Fermentations

All the MLF performed with *O. oeni* PSU-1 in WLM and in natural grape must were completed. Information about the development of AF in natural grape must can be found in Supplementary Figure S1.

The addition of mannoproteins in WLM did not change the duration or consumption rate of L-malic acid (Table 1). In all the conditions, MLF took 5 days to finish. As for the consumption rate, only the addition of 100 mg/L of mannoprotein extract showed a significant increase in this value (Table 1). Under the studied conditions, the hypothesized positive effect of mannoproteins on MLF [34] was not observed.

More differences were observed in natural grape wine MLFs. In these media, *O. oeni* showed a reduction in MLF length in *T. delbrueckii* and Sc3D wines with respect to the control ScQA23 wine (Table 2). The L-malate consumption rate increased by more than 25% in these wines. The MLF of *M. pulcherrima* wine was similar to the control condition. In general, the use of non-*Saccharomyces* promoted the MLF of *O. oeni* [10]. However, the fermentation with MpFlavia did not show this effect.

### 3.2. Mannoprotein Utilization

We studied mannoprotein utilization by *O. oeni* during MLF in wine by precipitating the total polysaccharide fraction and quantifying it as the concentration in mannose equivalents after acidic hydrolysis before and after MLF. This procedure allowed us to estimate the concentration of mannoproteins [37] that were degraded during MLF [34].

**Figure 1:**
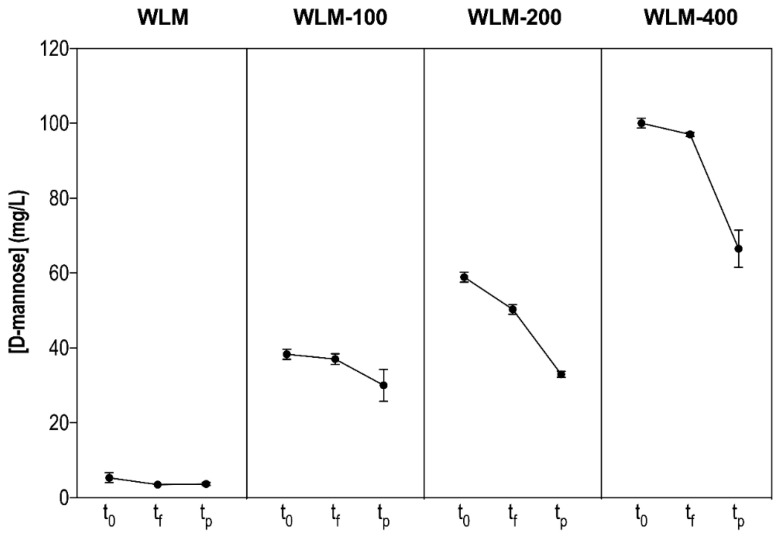
Mannoprotein concentration (mg of mannose eq./L) in wine-like medium (WLM) with mannoprotein extract addition throughout malolactic fermentation of *O. oeni* PSU-1. WLM-100, -200, and -400 represent the concentration (mg/L) of commercial mannoprotein extract added. t_0_, t_f_, and t_p_ represent before *O. oeni* inoculation, at the end of MLF ((L-malic acid) < 0.1 g/L), and after MLF (3 days after completion of MLF), respectively.

As regards WLM, a very low concentration of mannose equivalents (mannose eq.) was detected (Figure 1). This concentration was significantly increased by the addition of the commercial mannoprotein extract. We observed that the addition of this extract brought about a linear increase in the mannose concentration we quantified (data not shown). This corresponded to a mannose eq.: mannoprotein extract ratio (in mg/L) of around 0.23.

In all cases, the mannoprotein concentration detected decreased by the end of MLF (Figure 1). We also quantified it 3 days after completion of MLF to better understand the metabolism in WLM. In this post-MLF sampling we observed a dramatic reduction compared to the previous samplings (Figure 1). It seems that when L-malic acid is completely metabolized, the utilization of mannoproteins increases. As a result, we can relate the utilization of mannoproteins to a survival metabolism that is enhanced when the preferred substrate of *O. oeni* in wine, L-malic acid, is drained. It is also notable that this decrease was related to the initial mannoprotein concentration. The higher the initial mannoprotein concentration, the higher the degradation in this post-MLF sampling (Figure 1).

**Figure 2:**
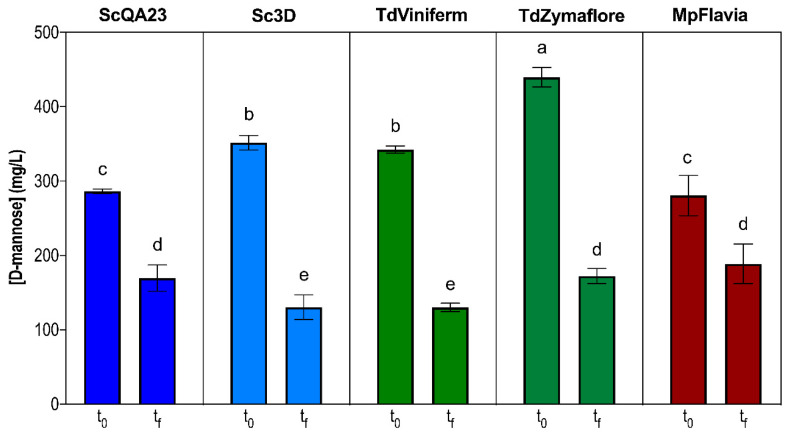
Mannoprotein concentration (mg of mannose eq./L) before and after malolactic fermentation in natural grape wines produced following different yeast inoculation strategies. Sc, Td, and Mp represent *S. cerevisiae*, *T. delbrueckii,* and *M. pulcherrima,* respectively, followed by the name of the commercial strain. t_0_ and t_f_ represent before *O. oeni* inoculation and at the end of MLF ((L-malic acid) < 0.1 g/L). ^a–e^ Significantly different at *p* ≤ 0.05 according to a Tukey post-hoc comparison test.

The results for mannoprotein utilization during MLF in natural grape wine can be found in Figure 2. Considering ScQA23 wine as the control condition, we observed a significant increase in mannoprotein concentrations in *T. delbrueckii* wines and when inoculating Sc3D. Similar values of around 340 mg /L of mannose eq. were quantified in TdViniferm and in Sc3D wines. As regards TdZymaflore wine, the mannoprotein concentration detected was the highest: 440 mg/L eq. of mannose on average. Thus, *T. delbrueckii* is a non-*Saccharomyces* yeast related to an increase in mannoprotein concentrations [19,20,37] in its fermented wines and also when added as yeast lees in a synthetic medium [34]. In addition, Sc3D is a selected commercial strain that is an overproducer of these macromolecules [20].

In *T. delbrueckii* and Sc3D wines, in which the concentration of mannoproteins after AF and mannose utilization by *O. oeni* were the highest (Figure 2), the L-malic acid consumption rate was also the highest (Table 2). We can therefore relate better MLF performance to enhanced mannoprotein utilization. It is interesting to note that the same *O. oeni* strains exhibited different use patterns depending on the yeast inoculated and the amount of mannoprotein released during AF.

In the case of MpFlavia, however, no differences were observed with respect to the control (280 mg/L of mannose eq. on average) even though this yeast—and this strain in particular—usually increases the mannoprotein concentration in wines [20,37] or produces similar concentrations to *S. cerevisiae*. The interactions of the yeast with the medium in which it is fermenting presumably determine the release of mannoproteins, probably due to the particular autolytic process in that medium [38]. In addition, *M. pulcherrima* and *S. cerevisiae* wines showed the lowest L-malic acid consumption rate (Table 2), which is also related to less mannoprotein release and utilization by *O. oeni*, supporting the idea that better MLF performances may be related to greater mannoprotein degradation (Figure 2).

The use of mannoproteins was different in the two types of wine studied. In natural grape wines, the highest mannoprotein degradation was detected once the L-malic acid was exhausted. This is probably because mannoprotein concentration is higher in natural wines and also because other inhibitor compounds, such as yeast metabolites or less nutrients, may be present in these wines. In fact, the yeast assimilable nitrogen (Table 2) in natural wines was less than half of that in the WLM (around 110 mg N/L). Under these more limiting conditions, the use of mannoproteins is enhanced in *O. oeni*. Thus, the breakdown of mannoproteins would release amino acids, apart from mannose, which could eventually be used as a nitrogen source by *O. oeni*.

### 3.3. General Wine Chemical Compounds

The MLF undergone with *O. oeni* in WLM transformed L-malic acid into L-lactic acid analogously in all wines (data not shown). We analyzed the chemical composition at the end of MLF and after three days of the completion of MLF (Table 1). No differences were observed within the conditions and sampling points as regards pH. Sugar concentration decreased slightly as a consequence of MLF. Nevertheless, the behavior in all WLM was similar and no sugar decrease was observed in the post-MLF sampling. Similarly, citric acid was not consumed by *O. oeni* in these conditions, neither at the end of MLF nor post-MLF. Citric acid decreased slightly at the post-MLF sampling point as a consequence of the consumption of the main carbon source—L-malic acid—being drained. The consumption of citric acid was around 0.1 g/L in all cases (Table 1) and no significant changes were detected in acetic acid concentration, as this is usually related to sugar or citric acid consumption. D-lactic acid, which can be an end product of sugars such as glucose, fructose, or mannose—which we can also relate to mannoprotein metabolism—was detected in higher concentrations in those wines to which the mannoprotein extract was added. Even when the concentrations were low after MLF, the addition of the mannoprotein extract resulted in a doubling of the D-lactic acid detected (Table 1). Moreover, in the post-MLF sampling the concentrations increased in all wines, whereas no significant increase was observed in the WLM without the addition.

More differences were observed in the compounds studied in the natural wines (Table 2). First, all the wines finished AF with similar L-malic acid concentrations of around 1.5 g/L (data not shown). The *T. delbrueckii* wines produced the most acidic wines, which were significantly different from the *M. pulcherrima* wine. The pH differences were as high as 0.1. As a result of MLF, the pH value increased, and the observed differences were also similar since they had similar amounts of L-malic acid. The residual sugar concentration (glucose + fructose) after AF was always below 2 g/L. Nevertheless, the wine inoculated with ScQA23 completely drained the sugars, whereas in the others that were fermented with the other *S. cerevisiae* strain and sequential inoculations with non-*Saccharomyces*, sugar traces were detected. These residual sugars were slightly consumed by *O. oeni* by the end of MLF in those wines inoculated with non-*Saccharomyces*, particularly those fermented with *T. delbrueckii*. In these wines the citric acid concentration after AF was slightly different as a result of the yeasts’ metabolism.

As mentioned earlier, the yeast assimilable nitrogen in the natural wines (Table 2) was lower than in WLM (110 mg N/L). The concentration of NOPA was significantly altered as a result of AF inoculation in the natural wines (Table 2). The *T. delbrueckii* and Sc3D wines had lower NOPA concentrations than the ScQA23 and *M. pulcherrima* wines. This can be explained by the different amino acid consumption patterns and preferences [39], which are also the result of yeast–yeast interactions [40]. However, the differences were small, and all the wines had enough NOPA concentration to ensure MLF. In contrast, similar ammonium concentrations were also observed (Table 2).

The use of non-*Saccharomyces* is usually related to higher citric acid concentrations after AF [10], although this has only been clearly observed with *Starmerella bacillaris* [41]. In the present study, the wines fermented with *T. delbrueckii* showed a significant increase in citric acid concentrations compared to the *S. cerevisiae* control wine. After MLF, this concentration was reduced as a consequence of the consumption by *O. oeni*. All the wines had around 0.11 g/L of this acid after MLF, with the exception of the ScQA23 wine, which had twice the concentration (Table 2).

After AF, the acetic acid concentration was different in the obtained wines. As described in the literature, the use of some non-*Saccharomyces* can change the concentration of this compound [14,23]. Generally speaking, *T. delbrueckii* tends to decrease it while *M. pulcherrima* increases it. In our study, *M. pulcherrima* significantly increased the volatile acidity of the wine after AF (up to 0.59 g/L on average), and only TdViniferm decreased it significantly (0.17 g/L on average). As a result of the consumption of sugars and citric acid by *O. oeni*, acetic acid and D-lactic acid were produced. After MLF, the concentration of acetic acid also depended on the combination of yeast species used in AF. The *S cerevisiae*-fermented wines had the intermediate concentration of acetic acid, while the *T. delbrueckii* wines had the lowest and *M. pulcherrima* had the highest.

D-lactic acid is a sugar related to the LAB metabolism and, therefore, it was not detected after AF. It increased as a result of the *O. oeni* metabolism after MLF (Table 2). The detected concentration was dependent on the mannoprotein concentration after AF (Figure 2), which resulted in higher D-lactic acid in the *T. delbrueckii* and Sc3D wines, 0.29 and 0.26 g/L on average, respectively.

### 3.4. Transcriptional Response of Mannose-Related Genes

**Figure 3:**
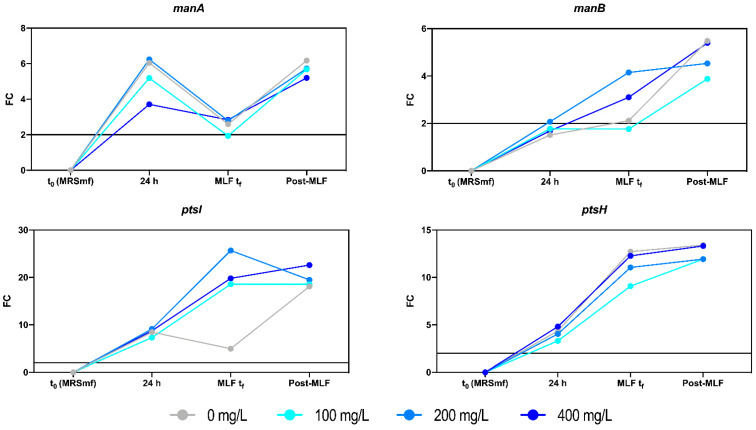
Evolution of relative expression as fold change (FC) of *manA*, *manB*, *ptsI,* and *ptsH* in WLM with different additions of mannoprotein extract, using the expression of the inoculum as the reference condition. FC = 2 is shown in the graph as the threshold for considering a gene to be upregulated.

The transcriptional regulation of four selected genes (Supplementary Table S1) previously related to mannose uptake in *O. oeni* [30] were evaluated in a wine-like medium with increasing concentrations of mannoprotein extract. This transcriptional regulation was compared to the expression of *O. oeni* prior to inoculation to determine the expression level of these genes under oenological conditions. The relative expression (RE) of the genes was quantified 24 h after inoculation, at the end of MLF, and 3 days after completion of the fermentation (Figure 3).

Studying the gene expression of mannose uptake-related genes under wine conditions is difficult since their expression is also dependent on the concentrations of other sugars or sugar alcohols [30,31,42]. These genes are generally activated when growing in the presence of sugars [31] and under ethanol stress [30]. Their relationship with the pH is more variable. Jamal et al. [30] observed that *manA* was expressed more in acidic conditions, whereas *manB* was more active in neutral pH, and *ptsI* and *ptsH* showed no variation.

In our study, the genes *manA*, *ptsI,* and *ptsH* were upregulated in response to WLM conditions 24 h after inoculation. The expression of *manB* did not show any change compared to the control condition (before inoculation). The expression at the end of MLF was variable depending on the gene and mannoprotein concentration. However, there was a general increase after the end of MLF in all the studied conditions. Three days after L-malic acid exhaustion, when most of the mannose consumption was detected, all the genes were upregulated compared to the control condition. It seems that when not many carbon sources are available, *O. oeni* increases the expression of these permeases to enable bacterial survival. Indeed, the bacterial population remained stable at around 10^7^ CFU/mL 3 days after the completion of MLF (data not shown). This highlights the importance of using alternative energy sources after L-malic consumption in order to allow for bacterial survival at least 3 days after completion of MLF.

**Figure 4:**
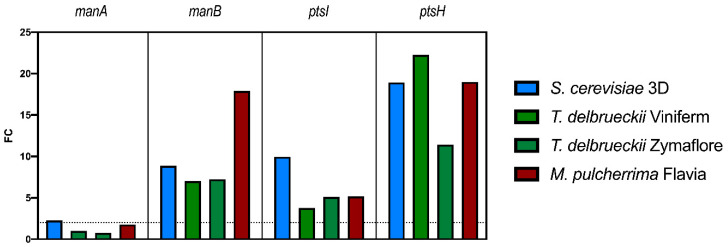
Relative expression as fold change (FC) of *manA*, *manB*, *ptsI,* and *ptsH* in natural wines fermented with different yeast inoculation regimes by the end of malolactic fermentation, using the expression in *S. cerevisiae* QA23 wine as the reference condition. FC = 2 is shown in the graph as the threshold for considering a gene to be upregulated.

We also studied these four genes in wine from natural grape must with different AF inoculation regimes by the end of MLF (Figure 4). Taking the expression of these genes in ScQA23 wine as the reference conditions, *manB*, *ptsI,* and *ptsH* were upregulated in the other wines. The gene *manA* did not show an expression pattern different from the reference condition with the exception of a slight upregulation in Sc3D wine.

It is interesting to see that the expression of these genes was upregulated in *M. pulcherrima* wine (Figure 4), which had low mannoprotein degradation levels (Figure 2), comparable to the reference condition. Therefore, the wine matrix must have an important effect on the expression of these genes, which makes it difficult to find any relation between RE and mannoprotein use. Moreover, it has to be remembered that these genes encode non-specific hexose permeases, which are usually active [30] and respond not only to mannose. Thus, it should not be surprising that these genes may be upregulated in response to oenologically stressful conditions, related with yeast inhibitory metabolites for *O. oeni*. Besides, higher REs were observed in these genes when *O. oeni* was fermenting in natural grape-derived wines with respect to WLM (Figure 3 and Figure 4). Nevertheless, no correlation was found between the RE and mannoprotein use patterns in *O. oeni*. This suggests a complex regulation dependent on the medium and not specifically linked to the expression of mannose uptake-related genes.

## 4. Conclusions

This study presents new information on mannoprotein utilization by *O. oeni* during MLF in wine-like medium and in wine. Different mannoprotein concentrations were quantified following an AF inoculation strategy. *T. delbrueckii* wines together with Sc3D wines were those with the highest concentrations of mannoproteins released. Mannoprotein utilization by *O. oeni* was dependent on the fermenting media. Low degradation of mannoproteins was observed when fermenting in a low mannoprotein concentration medium (WLM), whereas this degradation was higher in natural wines, with a higher mannoprotein content. This greater utilization of mannoproteins may be associated with a faster MLF in *T. delbrueckii* and Sc3D wines. The genes *manA*, *manB*, *ptsI,* and *ptsH*—directly related to mannose uptake but also active with other sugars—were upregulated in response to oenological conditions. *O. oeni* showed an increased RE of *manB*, *ptsI,* and *ptsH* in non-*Saccharomyces* and Sc3D wine compared to ScQA23 wine. Altogether, it seems that the mannoprotein metabolism is activated under wine conditions and that mannoprotein uptake is enhanced in stressful conditions. Further research is needed to clarify the regulation of mannoprotein metabolism in *O. oeni*, seeking out other possible genes/proteins involved in this metabolism. The use of different yeasts and mannoprotein extracts should be further evaluated with more *O. oeni* strains and conditions as potential activators of MLF.

## Figures and Tables

**Table 1:** Malolactic fermentation (MLF) parameters and concentration of various oenological compounds in wine-like medium. MLF and post-MLF refer to the sampling point at the end of MLF and 3 days of the completion of MLF, respectively.

	Consumption Rate (g/L·day) *	Duration (days)	pH		Glucose + Fructose (g/L)		Citric Acid (g/L)		Acetic Acid (g/L)		D-lactic Acid (mg/L)	
			MLF	Post-MLF	MLF	Post-MLF	MLF	Post-MLF	MLF	Post-MLF	MLF	Post-MLF
No addition	0.48 ± 0.02 ^b^	5	3.60 ± 0.01 ^a^	3.61 ± 0.0 ^a^	1.63 ± 0.01 ^a^	1.63 ± 0.02 ^a^	0.43 ± 0.03 ^a^	0.27 ± 0.08 ^a^	0.31 ± 0.01 ^a^	0.31 ± 0.01 ^a^	20.2 ± 1.2 ^b^	22.8 ± 0.6 ^b^
100 mg/L	0.55 ± 0.02 ^a^	5	3.60 ± 0.02 ^a^	3.61 ± 0.01 ^a^	1.63 ± 0.03 ^a^	1.63 ± 0.03 ^a^	0.41 ± 0.03 ^a^	0.31 ± 0.02 ^ab^	0.33 ± 0.01 ^a^	0.32 ± 0.01 ^a^	40.5 ± 7.3 ^a^	49.5 ± 1.3 ^a^
200 mg/L	0.49 ± 0.02 ^b^	5	3.59 ± 0.01 ^a^	3.61 ± 0.01 ^a^	1.65 ± 0.06 ^a^	1.68 ± 0.02 ^a^	0.43 ± 0.03 ^a^	0.35 ± 0.04 ^ab^	0.30 ± 0.01 ^a^	0.33 ± 0.01 ^a^	37.9 ± 9.0 ^a^	51.8 ± 1.0 ^a^
400 mg/L	0.48 ± 0.02 ^b^	5	3.61 ± 0.01 ^a^	3.61 ± 0.01 ^a^	1.65 ± 0.02 ^a^	1.68 ± 0.04 ^a^	0.45 ± 0.03 ^a^	0.37 ± 0.04 ^b^	0.29 ± 0.01 ^a^	0.32 ± 0.02 ^a^	38.5 ± 3.2 ^a^	56.1 ± 4.5 ^a^

^a,b^ Values are significantly different at *p* ≤ 0.05 according to a Tukey post-hoc comparison test. * Calculation based on consumption rate of L-malic acid (MLF) considering the period of exponential decrease of this compound.

**Table 2:** Malolactic fermentation (MLF) parameters and concentration of various oenological compounds in natural grape wines. AF and MLF refer to the sampling point after alcoholic fermentation and MLF, respectively. YAN means yeast assimilable nitrogen.

	Consumption Rate (g/L·day) *	Duration (Days)	YAN (AF)		pH		Glucose + Fructose (g/L)		Citric Acid (g/L)		Acetic Acid (g/L)		D-Lactic Acid (g/L)	Ethanol (% *v*/*v*)
			NOPA	NH_4_	AF	MLF	AF	MLF	AF	MLF	AF	MLF	MLF	AF
ScQA23	0.53 ± 0.02 ^b^	4	34.93 ± 6.90 ^ab^	17.67 ± 1.53 ^a^	3.49 ± 0.04 ^ab^	3.77 ± 0.04 ^d^	n.d.^a^	n.d.^b^	0.59 ± 0.02 ^ab^	0.21 ± 0.02 ^b^	0.29 ± 0.02 ^b^	0.70 ± 0.01 ^b^	0.20 ± 0.01 ^b^	10.8 ± 0.2 ^a^	
Sc3D	0.80 ± 0.02 ^a^	2	24.36 ± 0.76 ^b^	13.67 ± 2.08 ^a^	3.47 ± 0.01 ^ab^	3.67 ± 0.01 ^bc^	1.66 ± 0.48 ^b^	1.67 ± 0.07 ^a^	0.62 ± 0.03 ^b^	0.11 ± 0.01 ^a^	0.28 ± 0.04 ^b^	0.66 ± 0.00 ^b^	0.26 ± 0.06 ^cd^	11.0 ± 0.2 ^a^	
TdViniferm	0.73 ± 0.02 ^a^	2	23.71 ± 2.68 ^b^	16.00 ± 2.00 ^a^	3.42 ± 0.01 ^a^	3.59 ± 0.01 ^a^	1.05 ± 0.26 ^ab^	0.47 ± 0.38 ^a^	0.69 ± 0.00 ^c^	0.11 ± 0.02 ^a^	0.25 ± 0.04 ^ab^	0.47 ± 0.04 ^a^	0.29 ± 0.01 ^d^	11.2 ± 0.2 ^a^	
TdZymaflore	0.76 ± 0.02 ^a^	2	28.59 ± 5.09 ^ab^	13.67 ± 1.15 ^a^	3.44 ± 0.02 ^a^	3.62 ± 0.01 ^ab^	1.55 ± 0.14 ^ab^	0.61 ± 0.21 ^a^	0.73 ± 0.02 ^c^	0.11 ± 0.02 ^a^	0.17 ± 0.04 ^a^	0.43 ± 0.07 ^a^	0.24 ± 0.00 ^c^	10.7 ± 0.2 ^a^	
MpFlavia	0.50 ± 0.02 ^b^	4	37.29 ± 2.85 ^a^	13.33 ± 1.15 ^a^	3.54 ± 0.04 ^b^	3.71 ± 0.01 ^c^	1.41 ± 0.01 ^ab^	1.12 ± 0.57 ^a^	0.56 ± 0.02 ^a^	0.11 ± 0.01 ^a^	0.59 ± 0.04 ^c^	0.74 ± 0.01 ^b^	0.14 ± 0.01 ^a^	10.8 ± 0.2 ^a^	

^a–d^ Values are significantly different at *p* ≤ 0.05 according to a Tukey post-hoc comparison test. * Calculation based on consumption rate of L-malic acid (MLF) considering the period of exponential decrease of this compound. n.d.: not detected. D-lactic acid was not detected in wines after AF.

## Data Availability

Not applicable.

## Supplementary Materials

The following are available online at https://www.mdpi.com/article/10.3390/foods10071540/s1, Figure S1: Alcoholic fermentation dynamics where density decrease (grey) and yeast viability are represented for the used yeast species in each wine: *S. cerevisiae* (blue) and non-*Saccharomyces* (orange). Sc, Td and Mp represent *S. cerevisiae, T. delbrueckii* and *M. pulcherrima* respectively, followed by the name of the commercial strain. Values shown are the mean of triplicates ± SD, Table S1: Primers used in this work [43,44].
