# Supplementary material for: Use of Yeast Mannoproteins by Oenococcus oeni during Malolactic Fermentation under Different Oenological Conditions
Source: Foods. 2021 Jul 4;10(7):1540. doi: 10.3390/foods10071540 (PMC8305826; doi:10.3390/foods10071540)
Supplement: Supplementary file 1 [file foods-10-01540-s001.zip › foods-1269830-supplementary.pdf]

Supplementary Table 1. Primers used in this work.

Gene	Sequence (5'→3')	Reference
manA	F- TTCATTGGCGCAGCCGGTTT<br>R- GCCGTTGCTAAAATCGTCCC	[30]
manB	F- AGTCCAGTGGGCTTCTTTCT<br>R- TTGGTTCCAACGATTCAGGC	[30]
ptsI	F- GACGAACAGCTCATGCTTCG<br>R- ATCGATTAAGACCTGGCCGG	[30]
ptsH	F- CGATTACTGCTGACTCTGGC<br>R- TACCCGCGCCAAGACTCATT	[30]
dpolIII	F- AATTCGCACGGATTGTTTTC<br>R- GCGAACCAGCATAGGTCAAT	[43]
dnaG	F- TGTGGACGGAGTGGCAATGT<br>R- CAGTATTTTCTGTATATTTACTATCG	[44]
gyrA	F- CGCCCGACAAACCGCATAAA<br>R- CAAGGACTCATAGATTGCCGAA	[44]
gyrB	F- GAGGATGTCCGAGAAGGAATTA<br>R- ACCTGCTGGGCATCTGTATTG	[44]

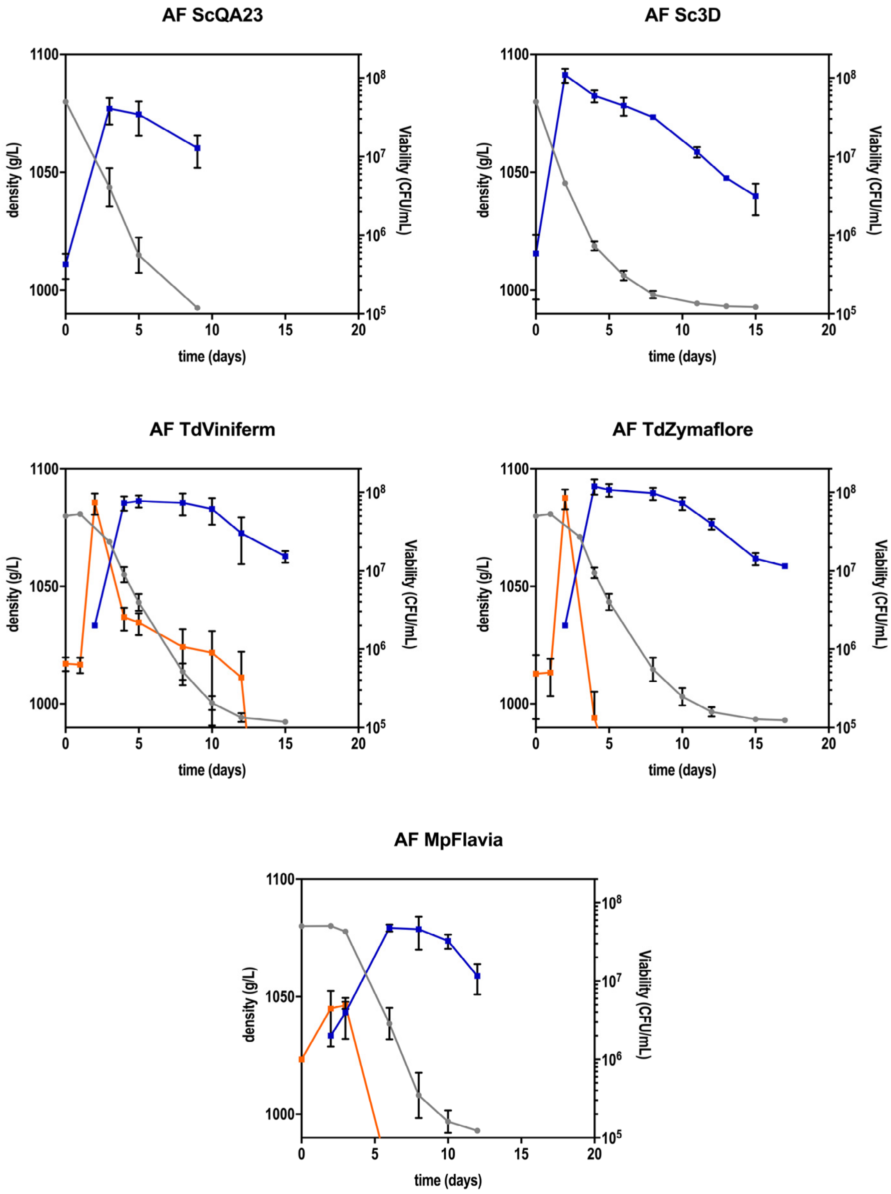

Supplementary Figure 1. Alcoholic fermentation dynamics where density decrease (grey) and yeast viability are represented for the used yeast species in each wine: *S. cerevisiae* (blue) and non-*Saccharomyces* (orange). Sc, Td and Mp represent *S. cerevisiae, T. delbrueckii* and *M. pulcherrima* respectively, followed by the name of the commercial strain. Values shown are the mean of triplicates ± SD.
